# Seroprevalence of SARS-CoV-2 in a Fully Operative Dentistry Academic Center in Madrid (Spain) During the De-escalation Phase of the COVID-19 Pandemic. Are Our Dentists at Greater Risk?

**DOI:** 10.3290/j.ohpd.b3464887

**Published:** 2022-10-19

**Authors:** Patricia Cintora, Rosa Rojo, Ana Martínez, Beatriz Ruíz, Juan Manuel Aragoneses

**Affiliations:** a Professor, Dental Center for Innovation and Advanced Specialties, Alfonso X el Sabio University, Madrid, Spain. Study concept, wrote the manuscript, final revision of the manuscript.; b Professor of Research Methodology, Faculty of Dentistry, Alfonso X el Sabio University, Madrid, Spain. Co-wrote the manuscript. final revision of the manuscript.; c Professor, Dental Center for Innovation and Advanced Specialties, Alfonso X el Sabio University, Madrid, Spain. Final revision of the manuscript.; d Director, Dental Center for Innovation and Advanced Specialties, Alfonso X el Sabio University, Madrid, Spain. Accessed and collected data, final revision of the manuscript.; e Dean, Faculty of Dentistry, Alfonso X el Sabio University, Madrid, Spain. Final revision of the manuscript.

**Keywords:** COVID-19, antibodies, seroprevalence, dental clinics, orthodontics.

## Abstract

**Purpose:**

To determine the prevalence of COVID-19 infection among dental professionals at an Academic Center in Madrid (Spain) at the beginning of the pandemic’s de-escalation phase.

**Materials and Methods:**

A cross-sectional study was designed. COVID-19 infection was determined by membrane-based immunoassay qualitative detection of IgG and IgM antibodies in human whole blood. Age, sex, race and professional qualification were recorded, as were symptoms compatible with COVID-19 infection whenever present. Data collected were analysed by means of descriptive and qualitative (X^2^) statistical analyses.

**Results:**

A total of 195 individuals were included (40 administrative professionals and 155 dentists). Seroprevalence at the end of the de-escalation phase was 20.0% among all the participants. The highest prevalence was found among the orthodontists (34.8%), followed by the paediatric dentists (28.6%) and oral surgeons (14.7%). Most subjects were positive for IgG and negative for IgM (79.5%).

**Conclusions:**

The seroprevalence of SARS-CoV-2 among dental professionals at the end of the de-escalation phase after the first wave of the pandemic was almost double the seroprevalence of the general population. Orthodontists had the highest rates of SARS-CoV-2 infection.

Spain is one of the European countries with more cases of Coronavirus disease 2019 (COVID-19), caused by Severe Acute Respiratory Syndrome-Coronavirus-2 (SARS-CoV-2), with more than 700,000 confirmed cases and more than 31,000 deaths by the end of September. Spain reached the peak of the coronavirus epidemic by March 20 and began decreasing one month later.^7^

Results of the third wave of the nationwide seroprevalence studies of SARS-CoV-2 in Spain in 61,075 participants have reported a 5% seroprevalence in the general population, with higher prevalence around Madrid (>10%) and in healthcare professionals (10.2% in healthcare, 7.7% in nursing homes or among other social workers and 6.4% in home caregivers).^17^

The spread of COVID-19 in the dental area must be controlled. Just as periodontal disease is associated with other disorders such as diabetes, liver disease, cardiovascular diseases, Alzheimer’s disease, rheumatoid arthritis, or even cancer,^10,11^ the scientific literature has shown that contracting COVID-19 could promote the development of lung cancer.^22^

Routine medical and dental clinical practice has been forced to adapt to the pandemic,^20^ including the development of new safety protocols for healthcare professionals. At the peak of the pandemic, dentists were instructed or advised (depending on the country) to stop providing treatment to dental patients except those with emergency complaints to protect dental professionals, their families, contacts, and their patients from the transmission of the virus, and also to conserve the supplies of personal protective equipment (PPE).^16^

Dental professionals are at high risk of becoming infected with COVID-19 due to the higher exposure to bioaerosols generated during dental procedures^8,24^ and the patient’s microscopic respiratory droplets (aerosol), still considered today the main path of transmission of COVID-19.^15^

To date, however, no specific studies have been performed to assess the prevalence of COVID-19 infection among professionals working in academic dental clinics compared to the general population. In addition, potential seroprevalence differences were expected to be found among the many dental subspecialties due to their different exposure to bioaerosols and small respiratory droplets.

The aim of this study was to determine the prevalence of COVID-19 infection among professionals from several areas of dentistry working at the Academic Center in Madrid, which deals with almost 500 patients per day, and compare it to the seroprevalence of COVID-19 infection among the general Spanish population at the beginning of the de-escalation phase (June 2020).

## Materials and Methods

The study was conducted according to the guidelines of the Declaration of Helsinki, and approved by the Ethics Committee of the Alfonso X El Sabio University (Madrid, Spain) (approval number 2/04/2020). All participants signed the informed consent for participation in the study.

This cross-sectional, observational study was carried out following the Strengthening the Reporting of Observational studies in Epidemiology (STROBE) recommendations.^23^

### Study Context, Participants and Variables

The study evaluated the seroprevalence for SARS-CoV-2 in the staff (administrative and dental personnel) of a University Dental Clinic (The Dental Center for Innovation and Advanced Specialties, Universidad Alfonso X el Sabio) located in Madrid (Spain), which serves approximately 40,000 patients per year. The group of dentists included professionals from the areas of oral surgery, orthodontics, endodontics, and paediatric dentistry.

Seroprevalence detection analyses were performed in workers returning to the clinic during the de-escalation phase (phase 1) after the confinement period, testing administrative staff on 11 May 2020 and the dental professionals on 2 and 5 June 2020). Those professionals who had been working during the confinement phase providing emergency services were also included in the seroprevalence study.

The following demographic variables were recorded: age, sex, race, and professional qualification in cases where symptoms were compatible with COVID-19 infection, as well as contact with suspected or confirmed cases and other risk factors whenever they were present.

### Detection of SARS-CoV-2 Antibodies

An immunochromatographic qualitative rapid test was applied to detect IgG and IgM antibodies in human whole blood. In the administrative staff, it was performed directly by finger prick to obtain a drop of blood (2019-nCoV IgG/IgM Rapid Test Cassette (Sienna, T and D Diagnostics Canada; Halifax, NS, Canada). Testing was performed immediately after the specimens were collected. For the rest of the staff, specimens were obtained through venipuncture by nurses and physicians from the clinic, then transported to and analysed at Laboratorios Echevarne (Madrid, Spain), using the same kit (Sienna).

A membrane-based immunoassay was used for qualitative differentiation between IgG and IgM against the 2019-nCoV antigen-coated particles in the test cassette, which yields results in 10 min. Seropositivity was considered as showing positive results in any of the three possibilities: positivity for IgG, positivity for IgM or positivity for both. The manufacturer reported a relative sensitivity of 100% for IgG and 85.0% for IgM, and a relative specificity of 98.0% for IgG and 96.0% for IgM, using RT-PCR as the gold standard.

### Sample Size

Other studies on detecting the seroprevalence of SARS-CoV-2 in health workers carried out in Spain during the first wave showed a prevalence between 2.8%^18^ and 10.3%.^2^ The binomial test was performed to compare a proportion taking as a reference a seroprevalence of 10.3% for a sample of 195 participants, with a power of 100%, and an alpha of 4%.

### Stastistical Analysis

In the statistical analysis, frequency tables, percentages and 95% confidence intervals (95% CIs) were obtained for categorical variables, while measures of central tendency and dispersion were calculated for continuous variables (mean, standard deviation [SD], standard error [SE], minimum and maximum, 95% CIs, median, and quartiles Q1 and Q3).

The X^2^ test was used to compare qualitative variables between groups. Univariate and multivariate logistic regressions (stepwise method) were performed to determine which factors were associated with seroprevalence. Finally, the Hosmer-Lemeshow test was performed to establish the goodness of fit of the models.

All the data were available from the health professionals of The Dental Center for Innovation, and Advanced Specialties in the first de-escalation phase were used.

In all cases, statistical significance was defined as p < 0.05, and the statistical analysis was performed using SPSS v.25.0 (IBM; Armonk, NY, USA).

## Results

A total of 195 participants were included, with the following profiles: administrative professionals (n = 40, 20.5%) and dentists (n = 155, 79.5%). In the dental group, the different specialties were stratified as follows: 11 endodontists (5.6%), 68 oral surgeons (34.9%), 69 orthodontists (35.4%) and seven paediatric dentists (3.6%). All participants were Caucasian, 120 were women (61.5%) and 75 were men (38.5%), with a mean age 32.15 ± 8.97 years (range: 23.51–70.25 years [Table 1]).

**Table 1:** Sociodemographic characteristics of subjects (n = 195)

	n	Total
		(n = 195)
**Age (years)**	**153**	
Mean ± SD		32.15±8.97
Min, max		23.51, 70.25
**Sex n (%)**	**195**	
Men		75 (38.5%)
Women		120 (61.5%)
**Professional profiles n (%)**	**195**	
Endodontist		11 (5.6%)
Orthodontists		69 (35.4%)
Paediatric dentists		7 (3.6%)
Students and professors		68 (34.9%)
Administrative staff		40 (20.5%)

SD: standard desviation.

The seroprevalence of SARS-CoV-2 in the whole sample was 20.0% (n = 39), with the highest prevalence in orthodontists (34.8%), paediatric dentists (28.6%) followed by the group of oral surgeons and (14.7%) endodontists (9.1%). The administrative staff had the lowest prevalence, with 5.0% (Table 2), with statistically significant differences among the different professional subgroups (p = 0.002). In the whole sample, the percentage of positive subjects was equal in women and men (20.0%).

**Table 2:** Seroprevalence in the studied population (n = 195)

	n	n (%)
Whole sample n (%)	195	39 (20.0%)
Professional profiles n (%)		
Endodontist	11	1 (9.1%)
Orthodontists	69	24 (34.8%)
Paediatric dentist	7	2 (28.6%)
Surgeons and implantologists	68	10 (14.7%)
Administrative staff	40	2 (5.0%)

Regarding the presence of IgG and IgM in seropositive subjects, most subjects were positive for IgG and negative for IgM (n = 31, 79.5%), while three subjects were negative for IgG and positive for IgM (7.7%) and five subjects were positive-positive (12.8%). Those with positive isolated IgG and no symptoms in the prior 2 weeks were allowed to return to work, whereas IgM or IgM/IgG individuals had sick-leave until showing a negative RT-PCR result or positive isolated IgG was achieved.

The multivariate logistic regression model showed that the professional profile ‘orthodontist’ in comparison with the administrative staff was a factor associated with seropositivity, with an OR (95% CI) of 10.133 (2.248–45.676) and p = 0.003 (Table 3 ). High OR values were also obtained for paediatric dentists (7.6), although without statistical significance (p = 0.067) (Table 3).

**Table 3:** The multivariate logistic regression model of factors associated with seropositivity

	OR 95% CI	p-value
Sex	0,486-2,057	1
Professional profile		0.060
Administrative	Reference	0.005
Endodontist	1.900 (0.156-23.135)	0.615
Orthodontists	10.133 (2.248-45.676)	0.003
Paediatric dentists	7.600 (0.867-66.593)	0.067
Surgeons and Implantologists	3.276 (0.680-15.783)	0.139

Among the group of COVID-19 seropositive dentists, mild symptoms were recorded in almost 25% of patients. The most relevant were fever and cough in 27.2% of cases, anosmia (loss of sense of smell) in 21.2% and ageusia (loss of sense of taste) in 15.2%. None of the seropositive individuals needed hospital treatment. A total of 27.2% of the seropositive subjects had symptoms compatible with COVID-19 infection, with fever being the most frequent symptom. Anosmia and ageusia were also frequent in seropositive subjects (21.2% and 15.2%, respectively).

## Discussion

There is no doubt that COVID-19 infection is influencing all aspects of life, particularly dental practice, where the risk of transmission is high. Key knowledge of relevant epidemiological data on the seroprevalence status among dental workers may be useful to develop efficient and reliable protocols, given the great incidence of asymptomatic cases and scarce access to PCR diagnostic tests during the peak of the pandemic.

To our knowledge, this is the first study of seroprevalence in dental clinics, otherwise the literature contains only a study^5^ based on a questionnaire survey which assessed the prevalence of symptoms and signs compatible with COVID-19 infection among dentists from Lombardy. In the whole sample, we reported a high level of seroprevalence (20.0%), similar to the rate of symptoms (14.4%) detected in dentists of Milan (Italy) with COVID-19 symptoms.^3^

We found that the seroprevalence in the first wave of the pandemic in Spain was around 2.8%^18^ among healthcare workers in a hospital on Mallorca and 10.3%^2^ among healthcare workers in the metropolitan area of Barcelona. Our study reveals a high seroprevalence among dental personnel.

These results also highlight the high degree of exposure of professionals to infected patients attending dental clinics before the lockdown phase in Spain, where no reliable data about the real incidence of the pandemic was available. Our results show that the seroprevalence of the dentists in our Institution was almost four times the rate of seroprevalence in the general population (5%), according to the National Seroprevalence Survey conducted in the first wave.

Statistically significantly different seroprevalence rates were found between dentists and the administrative staff working in the same building; this highlights the higher risk of transmission in a dental office environment, due to close contact with patients and the risk of cross-infection associated with dental procedures.^3,25^ For this reason, the safety measures to avoid contagion by SARS-CoV-2 include using personal protective equipment (PPE), gloves, goggles or protective screens, and FFP2 masks combined with surgical masks (changed every two hours), which should be set as a protocol in dental clinics.

Futhermore, the seroprevalence among the administrative staff (5.0%) was congruent with the seroprevalence reported in the Spanish population in the last seroprevalence study.^17^ That study also reported a general seroprevalence among healthcare professionals of 10.2%, whish was in the same range as the seroprevalence of endodontists in our study (9.1%). We also reported much higher rates in orthodontists (34.8%) and paediatric dentists (28.6%).

This statistically significant difference in seroprevalence between endodontists/surgeons/implantologists and orthodontists/paediatric dentists has been described here for the first time. Reasons for this difference could be attributed to different factors: more specific and protected procedures, fewer dental surfaces treated and less contact with patients in endodontics, surgery and implantology.

These results also put orthodontics and paediatric dentistry on alert regarding the risk of COVID-19 transmission as well as the risk of other aerosol-transmitted infectious diseases.^12^ Therefore, specific preventive measures for these specialities must be considered and implemented, such as specific disinfection procedures, specific PPE for the operators and specific measures for the patients.^16^ Other actions, as proposed recently, include increasing the use of digital orthodontics and the management of patients through “teledentistry”.^1^ In the case of orthodontics, these specific measures should be implemented and maintained during post-pandemic periods. While endodontists and paediatric dentists use more isolation measures, such as rubber-dam, and surgeons use more PPE and aseptic conditions relative to the armamentarium, in orthodontics, fewer protection measures are applied, while more people accompanying the patient are usually present during the visit.

The high seroprevalence observed in paediatric dentists should also be taken into account, in terms of avoiding the transmission of the virus to children as well as transmission from infected children to healthcare professionals.^14^ Recent evidence suggests that, although with mild or absent symptoms, the viral load in respiratory secretions from infected children can be as high as in adults; thus, children play an important role in virus transmission.^4^ Moreover, seroprevalence studies in Spanish children have been shown that children are just as likely as adults to be infected by another person.^19^ Further studies are needed to assess seroprevalence among different paediatric professionals in the healthcare settings.

Most seropositive subjects were positive for IgG and negative for IgM, thus indicating previous infection, while a minority of subjects were IgM positive, which could indicate the presence of the infection, in early, active and symptomatic stages.^21^ These results confirm that most transmission took place before the confinement phase, at the beginning of the first outbreak in Spain, when no specific measures had been taken yet.

Finally, in our study, we did not detect differences in seropositivity according to blood group, unlike other studies in which group A was associated with a higher risk for acquiring COVID-19 (confirmed by PCR) compared with non-A blood groups, and group O was associated with a lower risk in comparison with non-O blood groups.^9,12,13,26^ The reasons for not finding these associations could include our smaller sample and that serological testing could not detect all infected subjects, since it is estimated that in 20% of infected subjects, antibodies are not detected.^6^

In this context, we expect to perform further studies to assess the evolution of this population, for example, rates of re-infection, changes in Ig profiles, quantitative serological tests and rates of cases confirmed by PCR.

## Conclusions

Among professionals at a dental clinic, SARS-CoV-2 was more prevalent in orthodontists than in the rest of dental subdisciplines, with the lowest rates among the administrative staff. These results support specific protective measures for orthodontics in dental clinics.
